# *Mycobacterium tuberculosis Rv3802c* Encodes a Phospholipase/Thioesterase and Is Inhibited by the Antimycobacterial Agent Tetrahydrolipstatin

**DOI:** 10.1371/journal.pone.0004281

**Published:** 2009-01-26

**Authors:** Sarah K. Parker, Robert M. Barkley, John G. Rino, Michael L. Vasil

**Affiliations:** 1 Department of Pediatrics, University of Colorado Denver, Aurora, Colorado, United States of America; 2 Department of Microbiology, University of Colorado Denver, Aurora, Colorado, United States of America; 3 Department of Pharmacology, University of Colorado Denver, Aurora, Colorado, United States of America; University of Hyderabad, India

## Abstract

The cell wall of *M. tuberculosis* is central to its success as a pathogen. Mycolic acids are key components of this cell wall. The genes involved in joining the α and mero mycolates are located in a cluster, beginning with *Rv3799c* and extending at least until *Rv3804c*. The role of each enzyme encoded by these five genes is fairly well understood, except for *Rv3802c*. Rv3802 is one of seven putative cutinases encoded by the genome of *M. tuberculosis*. In phytopathogens, cutinases hydrolyze the waxy layer of plants, cutin. In a strictly mammalian pathogen, such as *M. tuberculosis*, it is likely that these proteins perform a different function. Of the seven, we chose to focus on *Rv3802c* because of its location in a mycolic acid synthesis gene cluster, its putative essentiality, its ubiquitous presence in actinomycetes, and its conservation in the minimal genome of *Mycobacterium leprae*. We expressed Rv3802 in *Escherichia coli* and purified the enzymatically active form. We probed its activities and inhibitors characterizing those relevant to its possible role in mycolic acid biosynthesis. In addition to its reported phospholipase A activity, Rv3802 has significant thioesterase activity, and it is inhibited by tetrahydrolipstatin (THL). THL is a described anti-tuberculous compound with an unknown mechanism, but it reportedly targets cell wall synthesis. Taken together, these data circumstantially support a role for Rv3802 in mycolic acid synthesis and, as the cell wall is integral to *M. tuberculosis* pathogenesis, identification of a novel cell wall enzyme and its inhibition has therapeutic and diagnostic implications.

## Introduction

**Figure 1:**
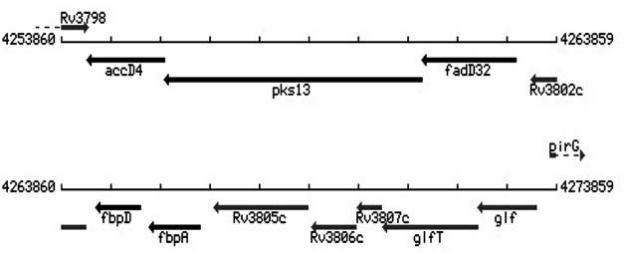
Mycolic acid synthesis gene cluster in *M. tuberculosis* H37Rv. Cluster is highly conserved in all actinomycetes.

**Figure 2:**
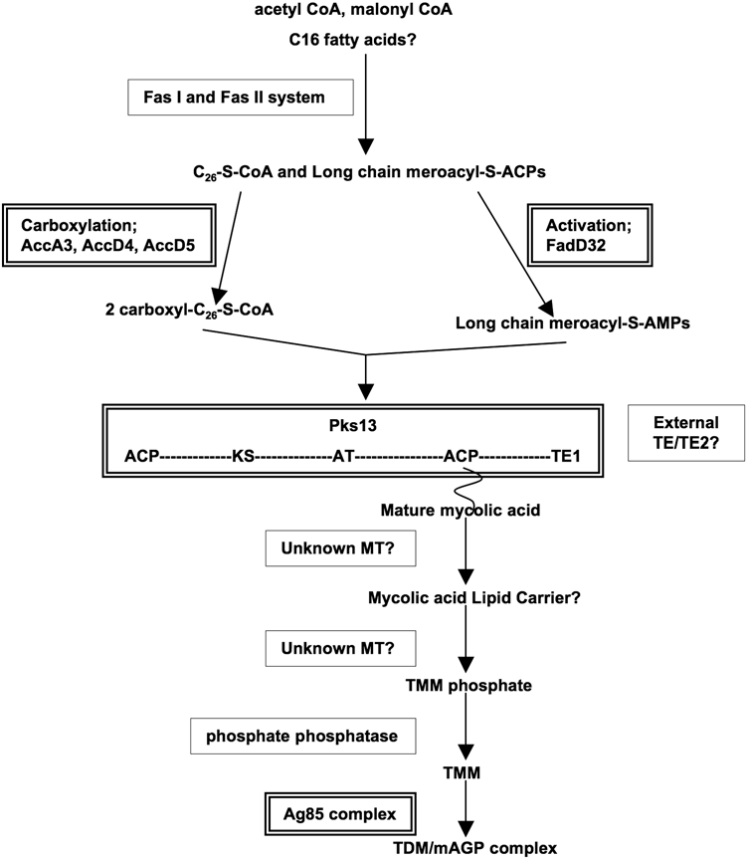
Approximate schematic of proposed mycolic acid synthesis. Fas I enzyme and FasII system elaborate the α and mero chains for nascent mycolic acids. Carboxylation by AccD4, AccA3 and AccD5 and activation by FadD32 convert these to their respective acyl-S-CoA and acyl-S-AMP forms. The AT (acyltransferase) domain of Pks13 attaches these via a tioester bond to the phosphopentathiene-modified ACP domains, and facilitates transfer to the KS (ketosynthase) domain. Via Claisen-type condensation and reduction, the two chains are joined to form a mature mycolic acid attached via a thioester to Pks13, with loss of CO_2_. The mature mycolic acid is then hydrolyzed from Pks13 by either the TE1 domain of Pks13, or an external TE. An external TE2 may function to unclog the Pks13 if it is mis-acylated. The liberated mycolic acid may be transferred to a lipid carrier, such as Myc-PL, via an unkown MT (mycolyltranferase); this may facilitate its transfer across the plasma membrane. Eventually it is tranferred by another MT to TMM. TMM is used as a mycolic acid donor for the acceptors of TMM, to form TDM, or AG, to form mAG. Double-boxed enzymes indicate step involves proteins encoded by genes in the mycolic acid synthesis gene cluster with *Rv3802c*. Question marks represent activities encoded by unknown enzymes and a possible role of Rv3802. Adapted from references [7], [13], [36], [44].

*M. tuberculosis* infects one third of the world, makes over 8 million ill each year, and kills 1.8 million, 450,000 of whom are children [1]. Though one of the oldest known human pathogens, our ability to combat spread of this disease remains insufficient and the global health burden of tuberculosis is increasing [2]. Key to the success of the tubercule bacillis is its uniquely complex lipid rich cell wall, and cell wall synthesis pathways are current target areas for drug development [3]–[6]. The cell wall of mycobacteria is considered a bilayer, and the lipids integral to the bilayer are the myolic acids. Just exterior to the cell membrane lies the mycolyl-arabinogalactan-peptidoglycan complex (mAGP). This complex forms the stable scaffolding for the outer component of surface-exposed lipids and glycolipids, such as trehalose monomycolate (TMM) and dimycolate (TDM). The mycolic acids of these glycolipids are noncovalently intercolated with the mAGP. Mycolic acid containing lipids are not only essential for the survival of *M. tuberculosis*, they contribute to pathogenesis through a variety of mechanisms from cell signaling and host evasion to granuloma formation. The genes involved in joining the mero and α mycolate into a mature mycolic acid and transferring it to trehalose or arabinogalactan are located in a gene cluster from *Rv3799c* to at least *Rv3804c* (Figure 1). The role of each enzyme encoded by these five genes is fairly well understood, except for the product of *Rv3802c*, though these five genes do not account for all the necessary steps in the pathway [7]. A summary is presented in Figure 2. In brief, FadD32 (fatty acid dehydrogenase, *Rv3801c*) and AccD4 (*Rv3799c*), along with the other acyl-CoA carboxylases, AccA3 and AccD5, participate in deriving meroacyl-AMP (from FAS-II) and 2-carboxyl-C26-S-CoA (from FAS-I), respectively. These are attached via thioester bonds to phosphopentathienes on the acyl carrier domains of Pks13 (polyketide synthase, *Rv3800c*), a multidomain protein which, via Claisen condensation, creates a mature mycolic acid. A reduction step is needed and one enzyme so far, CmrA, has been identified for this role [8]. The deposition and export of the mycolates is less well understood. A mature mycolic acid is eventually transferred either to arabinogalactan (to form mAGP) in part by the Ag85 proteins, or to an α,α-trehalose-6-phosphate (T6P, leading to TMM) by an unknown mycolyltransferase [9]. A phospholipid carrier, 6-0-mycolyl-a-D-mannopyranosyl-1-monophosphoheptaprenol (Myc-PL), is reported to be involved in *Mycobacterium smegmatis* [10]. For TDM formation, a second mycolic acid is added to TMM by the antigen 85 complex mycolyltransferases, including Ag85A (*Rv3804c*) [11]. In this study we investigated the possible contributions of the *Rv3802c* gene product to the mycolic acid synthesis pathway. Our data demonstrate that the products encoded by *Rv3802c* and the non-orthologous but homologue *MSMEG_1403* possess both phospholipase A (PLA) activity and thioesterase activity. These activities are consistent with a role in mycolic acid biosynthesis as this pathway involves multiple ester and thioester bonds. In addition, the *Rv3802c* gene product, but not that of the distant homologue *MSMEG_1403* in *M. smegmatis*, is inhibited by the known human fatty acid synthase thioesterase (FASTE) domain inhibitor, THL. THL has been demonstrated to decrease mycolic acid synthesis, leading to defects in the mycobacterial cell wall, though the targets of its antimycobacterial action were unknown. Though these lines of evidence are circumstantial, taken together with the genetic data, these findings support a role for Rv3802 in mycobacterial mycolic acid synthesis.

## Results

### Genome comparisons of the Rv*3802c* Locus

**Figure 3:**
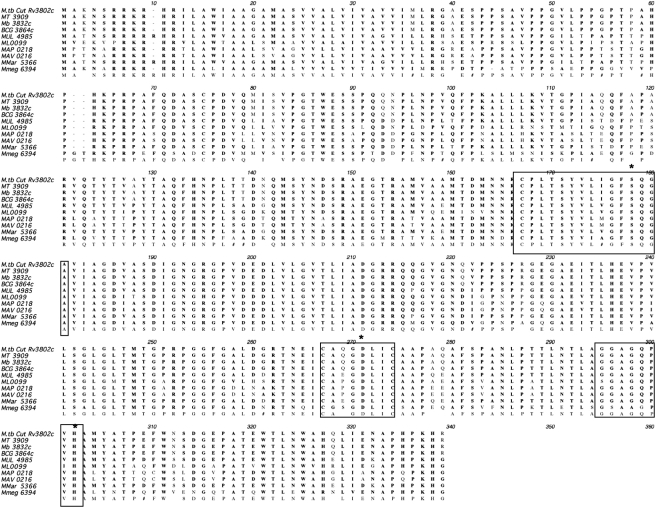
Clustal W alignment of Rv3802 with its orthologs in mycobacteria demonstrating high conservation. Cutinase motif is boxed with asterisk over putative catalytic site amino acids.

We analyzed a 30-kb chromosomal region surrounding *Rv3802c* in *M. tuberculosis H37Rv* and compared it to that of other mycobacteria and corynebacteria. *Rv3802c* is located in a mycolic acid synthesis gene cluster from *Rv3799c* to *3804c*, which encode AccD4, Pks13, FadD32, Rv3802, Ag85D and Ag85A, respectively (Figure 1). The cluster is conserved in all mycobacteria, corynebacteria, nocardia, and rhodococci with available annotated databases, indicative of functionality common to actinomycetes. Of the seven cutinases present in *M. tuberculosis*, *Rv3802c* is the only one reported to be essential, and is the only one conserved in *M. leprae*, which is considered a minimal genome [12], [13]. Essentiality was determined with Himar-1-based mutagenesis experiments in *M. tuberculosis* and *Mycobacterium bovis* BCG, and *Rv3802c* had an insertion/genomic probe ratio of 0.08 (cut-off for essentiality experimentally set below 0.2) [12]. However, this is still a screen and the essential nature of this gene needs to be confirmed with deletion/complementation studies. The three upstream genes encoding the AccD4, Pks13 and FadD32 proteins have similar insertion/genomic probe ratios (0.02–0.16) and were confirmed to be essential in *M. tuberculosis* and *M. smegmatis* [14], [15]. The amino acid sequence of Rv3802 is highly similar to that its orthologs in other mycobacteria (76–100%, Figure 3) and is 60% similar to the ortholog in *C. glutamicum*; it shares only 0–40% similarity with the other annotated cutinases in *M. tuberculosis*. Rv3802 is a 337 amino acid protein with a predicted secretion signal and an altered, but recognizable, cutinase motif [16]. The motif contains the classic S, D, H catalytic triad, though in Rv3802 and orthologs the probable catalytic H is 30 amino acids from the D rather than the 12 described in the classic motif[16]. This motif places Rv3802 and the other cutinases in the α/β-hydrolase fold family, in company with known mycolyltransferases (Ag85 complex), known PLAs and known thioesterases. Like all α/β-hydrolase fold enzymes, the predicted structure has an oxyanion hole, which provides a positively charged hole to stabilize the transient oxyanions formed during acylation and deacylation, and an acyl binding pocket lined with hydrophobic residues [17], [18]. Rv3802 is predicted to be a membrane protein, though it is unclear if it is on the cytosolic or extracytosolic side[19]. It has only one predicted membrane spanning helix located within the putative secretion signal; the GRAVY value with and without the secretion signal are −0.15 and −0.29, respectively, implying moderate solubility, though indeed experimentally it has been found in the membrane fraction [20], [21].

### Expression and purification of recombinant proteins *M. tuberculosis* Rv3802, its mutants, and *M. smegmatis* mc^2^155 MSMEG_1403 in *E. coli*

**Figure 4:**
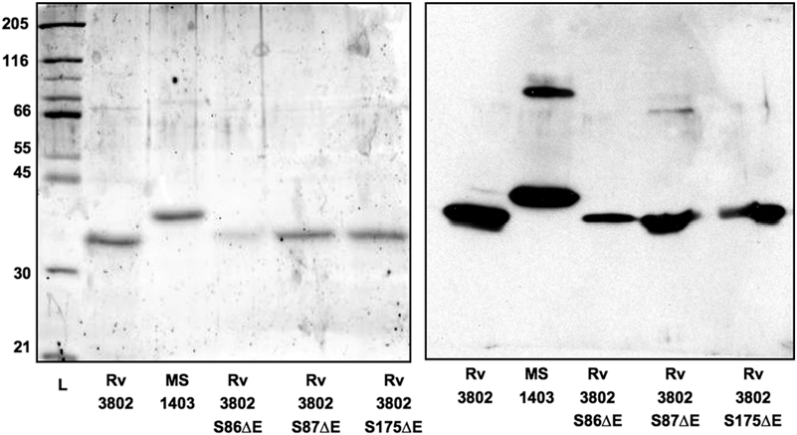
Products of Rv*3802c* and *MSMEG_1403* expression in *E. coli*. Left: polyacrylamide gel visualized with Sypro Orange™. Right: immunoblot with streptavidin antibody.

The genes *Rv3802c* and *MSMEG_1403* were PCR-amplified from their respective genomes without their secretion signals and placed under the control of the IPTG inducible T7 promoter in the expression vector pET23a. The vector derived 6× His tag was not used in lieu of an engineered streptavidin tag, and expression in each recombinant strain was verified using an anti-streptavidin antibody (Figure 4). The product of *MSMEG_1403* is not the ortholog of Rv3802, rather it is a homologous protein we purified from *M. smegmatis* culture supernatant for its PLA activity [22]. Though not the focus of this manuscript, we include information on MSMEG_1403 here because it confirms that cutinases have PLA and TE activity, novel activities for this class of enzymes. In addition, it demonstrates that a non-orthologous cutinase, MSMEG_1403, is not inhibited by THL, which is significant because growth of *M. smegmatis* in culture also is not inhibited by THL [23]. Amino acid changes in Rv3802 were made in serines 86, 87 and 175. Each was changed to a glutamic acid. Ser175 is predicted to be the catalytic serine. Either Ser86 or Ser87 is predicted to stabilize the oxyanion hole, based on publications with *F. solani* cutinase Ser42; these were mutated with the goal of finding a partially active mutant [24]. Solubility was increased with use of sarcosyl for membrane disruption. Purity was assessed to be at least 95% on an SDS polyacrylamide gel stained with a technique sensitive to 10 ng of protein (Figure 4). Notably, MSMEG_1403 electrophoreses larger on the gel than Rv3802, though Rv3802 is predicted to be 32.5 kD and MSMEG_1403 30 kD. Reasons for this are unclear, but it is a consistent finding. In addition, with MSMEG_1403, dimers and trimers as well as monomers are often visible.

### Activity of purified proteins *M. tuberculosis* Rv3802 and *M. smegmatis* mc^2^155 MSMEG_1403 on various substrates

In order to understand its location in the cell wall pathway, various substrates and activities were explored. This included assays for esterase, lipase, phospholipase, thioesterase and mycolyltransferase activities.

**Figure 5:**
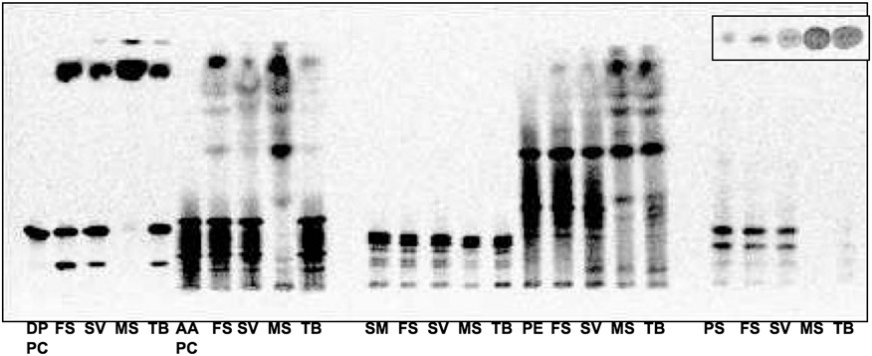
Hydrolysis of various phospholipid substrates by *F. solani* cutinase (FS), Snake venom PLA (SV), MSMEG_1403 and Rv3802, demonstrating hydrolysis of all except SM. Thin layer chromatography using ^14^C-labeled DPPC = dipalmitoyl phosphatidylcholine labeled on both fatty acids (FA), AAPC = phosphatidylcholine with ^14^C-arachidonic acid on the *sn*-2, SM = sphingomyelin ^14^C-labeled on headgroup, PE = phosphatidylethanolamine ^14^C-labeled on *sn*-2 FA, PS = phosphatidylserine ^14^C-labeled on headgroup. Inset is equivalent aqueous phase of phosphatidylserine assay.

Phospholipids are integral to both host membranes and the mycobacterial cell wall, and it is known that exogenous radiolabeled ^14^C-phospholipids are incorporated into the cell wall [25], [26]. In theory, this is through fatty acid incorporation into the FAS-II pathway, though complete catabolism may also occur. In any event, PLA activity is a necessary part of this process, thus we further investigated the PLA activity of purified Rv3802 and MSMEG_1403. Of note, we described PLA activity previously, but on partially purified native protein rather than purified exogenous protein [22]. To investigate the ability of Rv3802 and MSMEG_1403 to hydrolyze host and mycobacterial phospholipids, hydrolysis of ^14^C-radiolabeled phospholipids was assessed by TLC. Rv3802 and MSMEG_1403 were active in a PLA-type fashion (hydrolysis to respective lysophospholipid and fatty acid) on phosphatidylcholine, phosphatidylethanolamine, and phosphatidylserine, but not sphingomyelin (Figure 5). The enzymes were able to hydrolyze both palmitic acid and arachidonic acid containing phospholipids.

Because these proteins are annotated as cutinases, hydrolysis of nitrophenyl butyrate (NPB) was investigated as a surrogate for cutinase activity. This is essentially an esterase activity with colorimetric detection of the nitrophenyl group freed from NPB. Both enzymes were active esterases, though the turnover rates were relatively slow (Table 1).

**Table 1:** Kinetics of Rv3802 and Rv3802 S87ΔE mutant.

Enzyme and Substrate assayed	Km, mM	Vmax Moles[S] min^−1^ mg^−1^	Kcat sec^−1^	Specificity constant M^−1^ sec^−1^
**Rv3802**	23.52+/−3.78	1.62+/−0.08×10−8	8.81+/−0.31×10−3	0.37+/−0.01
**NPB**				
**Rv3802**	12.91+/−2.53	8.76+/−0.55×10−9	4.77+/−0.28×10−3	0.37+/−0.02
**S87ΔE**				
**NPB**				
**Rv3802**	0.017+/−0.003	1.35+/−0.04×10−7	7.33+/−0.62×10−2	37.00+/−11.08
**Palmitoyl-S-CoA**				
**Rv3802**	2.28+/−1.06	1.11+/−0.25×10−7	8.45+/−2.53×10−2	37.00+/−11.08
**Decanoyl-S-CoA**				

Rv3802 S86ΔE and S175ΔE are inactive in both assays, as is snake venom PLA_2_. NPB substrate represents an ester bond, while acyl-CoA substrates represent thioester bonds.

Though Rv3802 may function for hydrolysis only, evaluation of the mycolic acid synthesis pathway suggests a possible role as a transferase (Figure 2). Rv3802 and Antigen85A (Rv3804), a known mycolyltranferase, are both α/β-hydrolase fold enzymes. The acyl binding pocket of Ag85A functions to transfer the second mycolic acid onto TMM to from TDM. We therefore investigated acyltransferase activity of Rv3802 and MSMEG_1403. Various acyl donors were paired with acceptors in enzyme assays. First, to investigate transacylation of trehalose (similar to Ag85) ^14^C-trehalose was used as the acceptor, and cell wall lipids or crude cell wall extract (with lipid and protein) were used for donation; no transacylation was detected except with the positive control, Ag85C (data not shown). T6P is not currently commercially available, thus a similar assay was done with the acceptors mannose-6-phosphate and glycerol-6-phosphate, with no observed acylated product. These same acceptors were used but the donor changed to ^14^C-P-CoA (thioester bond) or ^14^C-DPPC (ester bond), and again no acylation occurred, though hydrolysis of the donors did occur. ^14^C-P-CoA and ^14^C-DPPC were again used as donors, with crude cell wall extract (lipids and proteins, ATP also added) used for the acceptor; no new acylated products emerged. These ^14^C-P-CoA and ^14^C-DPPC assays were repeated with the addition of crude cell wall extract alone and with cytosolic extract, with the hope that native donor or acceptor would be present, and again, no transacylation was observed. Transacylation assays were repeated several times with different amounts of donor, acceptor and enzyme, and different amounts of time from 1 to 72 H. The only result for Rv3802 and MSMEG_1403 in all assays was the release of fatty acid (not shown).

**Figure 6:**
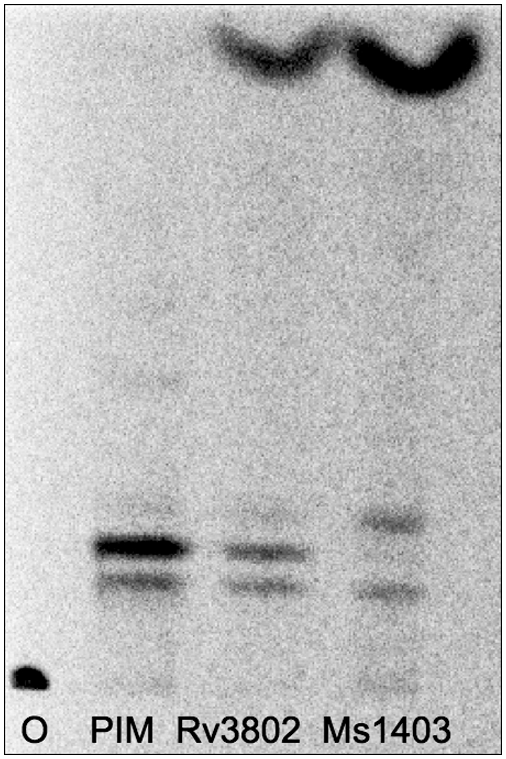
Hydrolysis of PIM purified on a waters column. Dot over O represents origin. Lane 1 is purified PIM control. Lanes 3 and 4 contain PIM plus Rv3802 and MSMEG_1403, respectively.

As a more global cell wall assay, *M. smegmatis* mc^2^ 155 cell wall was radiolabeled with ^14^C-acetate, and the outer cell wall was extracted as in methods. This labeled cell wall was assayed with Rv3802 and MSMEG_1403 protein, and diminution of a labeled lipid as well as appearance of new lipid with a similar retention factor (Rf) to known ^14^C-palmitic acid resulted. Mass spectrometry of the apparent substrate revealed it as phosphatidylinositol mannoside 2 (PIM2); this was purified by normal phase chromatography on a silica column, and hydrolysis by Rv3802 and MSMEG_1403 confirmed (Figure 6). Based on Rf in this solvent, the PIM hydrolysis product is consistent with a fatty acid, including tuberculostearic acid[27]. This result was not surprising given that we have demonstrated that these enzymes possess PLA activity.

We investigated the thioesterase activity of these enzymes because of the possibility that Rv3802 and its orthologs hydrolyze the thioester bond of nascent mycolic acids on Pks13 as the terminal event in synthesis or, alternatively, that the *Rv3802c* gene product could be a “proof reading” thioesterase. This assay uses acyl-CoA as the thioester substrate, with colorimetric detection of TNB^2−^ formed when DTNB reacts with the hydrolyzed product HS-CoA[28]. Both *M. tuberculosis* Rv3802 and *M. smegmatis* MSMEG_1403 protein were active as thioesterases (Table 1).

For the kinetic analysis of Rv3802, the time-dependent hydrolysis of palmitoyl CoA (P-CoA) and decanoyl CoA (D-CoA) was measured for thioesterase activity, and of NPB for esterase activity (see Table 1). These kinetic values are similar to those obtained with other thioesterases. While Rv3802 appears to favor thioester bonds over ester bonds considering the kinetic values, the difference in acyl chain length may account for this, as Rv3802 has a higher affinity for hexadecanoyl than decanoyl thioesters. A comparison between similarly acylated thioesters and esters would be of interest, but we are limited by available substrates and solubility of compounds, as the nitrophenyl esters are less soluble in aqueous solvents than CoA thioesters, and thus one would end up comparing a soluble substrate to a micellar substrate, which may influence results [29]. Rv3802 was not active on acetyl-CoA. The Rv3802 mutants of S175ΔE and S86ΔE were essentially inactive, while the S87ΔE mutant is partially active in both assays (Table 1). Snake venom PLA_2_ was not active in either the colorimetric esterase or thioesterase assays (data not shown).

### Inhibition of *M. tuberculosis* Rv3802 and non-inhibition of *M. smegmatis* mc^2^155 MSMEG_1403 with THL

**Figure 7:**
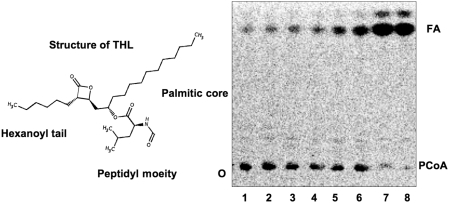
TLC demonstrating thioesterase activity of Rv3802 on ^14^C-labeled P-CoA and its inhibition by THL, structure above (adapted from DrugBank, http://www.drugbank.ca/drugs/DB01083). Lanes: 1) ^14^C-P-CoA alone, 2–8) serially dilutions decreasing the amount of THL from 12.5–0 µM THL with 3.0 µg of Rv3802. Origin is indicated with an O, start substrate indicated with P-CoA, hydrolysis product indicated with FA (fatty acid).

THL is reported to inhibit the thioesterase domain of human FAS, thus we investigated the ability of THL to inhibit *M. tuberculosis* Rv3802. Inhibition of PLA activity is demonstrated in Figure 7. The kinetics of inhibition were performed using the thioesterase assay. Complete inhibition was seen at THL concentration of 3.125 µM, a molar excess of 6.7∶1 inhibitor to enzyme. The apparent Ki and IC_50_ for THL against Rv3802 are 5 and 250 nm, respectively, which are lower than against human FASTE, for which the apparent Ki and IC_50_ are reported at 100 nm and 1.35±0.34 µM, respectively [30]. It is significantly better than the 200∶1 molar excess needed for partial inhibition reported against the only reported mycobacterial target of THL, Rv0183[30]–[32]. Interestingly, THL did not inhibit MSMEG_1403 up to THL concentrations of 25 µM, a 500 M excess.

## Discussion

In this report we described the heterologous expression, purification and enzymatic characterization of an annotated cutinase, Rv3802, from a mammalian pathogen. Though the proteins encoded by *M. tuberculosis* cutinases, including Rv3802, have been immunologically characterized, their function in organisms that do not encounter cutin has not been elucidated [33]. We previously attributed mycobacterial PLA activity to the cutinases, and in this manuscript additionally ascribe thioesterase activity. Logical potential uses for these activities by a mammalian pathogen include host lipid hydrolysis for carbon scavenging, or mycobacterial cell wall construction/remodeling. As there are seven annotated cutinases in *M. tuberculosis*, it may be they serve a variety of functions. The function of *Rv3802c* and its orthologs is suggested by its location in a gene cluster for mycolic acid synthesis. In addition, the importance of *Rv3802c* is supported by its putative essentiality, its ubiquitous presence in actinomycetes, and its unique conservation in the minimal genome of *M. leprae*. To date, all of the genes surrounding *Rv3802c* are demonstrated to serve vital functions in mycolic acid synthesis. This genetic data implying a role in mycolic acid synthesis is further supported by Rv3802's: (i) membership in the α/β-hydrolase fold family with known PLAs, mycolyltransferases and thioesterases; (ii) possession of thioesterase activity; and (iii) enzymatic inhibition by a known mycolic acid synthesis inhibitor, THL.

The compound THL is sold over the counter as the weight loss agent Alli™. It inhibits pancreatic lipase so that long chain fatty acids cannot be absorbed. During activity based screening, THL was noted to be a selective inhibitor of the TE domain on human FAS [30]. FAS is a modular protein like Pks13. FAS has little effect on normal cells because they take up dietary lipids but, in tumor cells, FAS is upregulated and contributes to angiogenesis; thus THL is currently a lead compound in the development of anticancer drugs [28], [34]. This development is focusing on creating more absorbable and specific forms[30], [35]. The published apparent Ki and IC_50_ for THL against human FASTE are 100 nm and 1.35±0.34 µM, respectively[36]. THL has been demonstrated to inhibit *M. tuberculosis* growth at <30 µg/mL, decreasing the production of mycolic acids with concomitant defects in the cell wall, though the target(s) were undefined [23]. Possible targets of THL, including the diacylglycerol acyltransferase Rv3130 and the FAS-I and II pathways were investigated, but none were identified as a target[23]. A monoglyceride lipase, Rv0183, was later identified as a possible target, but a 200∶1 molar excess of inhibitor to enzyme was needed to see an effect[32]. Based on our knowledge that Rv3802 is an α/β-hydrolase fold with TE activity, and the structure of THL, we investigated the ability of THL to inhibit recombinant Rv3802 and found it to inhibit at nanomolar concentrations, as described. The mechanism of inhibition is assumed to be similar to the mechanism of human FASTE inhibition. For human FASTE, THL is demonstrated to inhibit competitively, with the palmitic core of THL fitting into the hydrophobic substrate binding pocket and the hexanoyl tail packing against the catalytic histidine, significantly delaying hydrolysis (Figure 7) [31], [34]. Eventually it is hydrolyzed, thus inhibition is reversible. The inhibition of an enzyme whose gene is located in the mycolic acid synthesis gene cluster, together with previously published observations that THL affects the cell wall and decreases mycolic acids, supports a cause and effect relationship between THL and Rv3802. Although we demonstrated that THL inhibits Rv3802 in assays, this does not confirm that Rv3802 is the primary target of THL in culture. It is likely that THL inhibits other enzymes. In any case, the knowledge that THL inhibits Rv3802 at least gives a framework for exploring inhibition of Rv3802 and other THL targets further in *M. tuberculosis*.

Though further investigation will be required to pinpoint the exact function of Rv3802, examination of the mycolic acid synthetic pathway suggests a role as a Pks associated thioesterase or as a mycolyltransferase. Though we could not demonstrate mycolyltransferase activity, our data is limited by the need to use surrogate substrates and acceptors, thus this remains a distinct possibility. There are multiple unknown mycolyltransferases required in the pathway, and similarities between the known mycolyltranferase Ag85 proteins and Rv3802 are hard to ignore. However, the unusual ability to hydrolyze thioesters strongly suggests some type of interaction with nascent mycolic acids while docked via thioester bonds on Pks13.

Interaction with Pks13 could be in the form of an exogenous type 1 thioesterase, (TE1) or as an exogenous type 2 thiosterase (TE2) [37]. Type I polyketide synthases (PKSs) are modular, “assembly line”-like enzymes responsible for the steps in the biosynthesis of polyketides and mycolic acids [38]. Pks13 in *M. tuberculosis* has all the necessary modules to produce a mature mycolic acid: one acyltranferase domain for loading and transfer of selected acyl chains to the phosphopentatheine (PP) binding motif of the acyl-S-carrier protein (ACP) domain; two ACPs for thioester binding, via a PP, of the meroacyl chain from FadD32 and the 2 carboxyl-C_26_ chain from AccD4, AccA3 and AccD5; a ketoacyl synthase domain for Claisen-type condensation; and one TE1 domain for chain termination and hydrolysis. The reduction event to form the mature mycolic acid may be catalyzed by more than one protein, but to date CmrA has been identified[8]. The mature mycolic acid can then be exported and transferred to the mycolate containing components of the mycobacterial cell wall, via unknown mycolyltranferases. However, it has been proposed that the TE1 on the C terminal domain of Pks13 does not liberate the mature mycolic acid because the presence of free mycolic acid would be unwieldy for the cell [7]. A solution to this problem is to have an external TE1 with mycolyltranferase activity. In theory, Rv3802 could fill this role, though admittedly we find no clear precedent for this in the literature.

An alternative role for this protein is that of the precedented TE2. TE2s are well described in association with Pks and NRPS modules in bacteria, fungi and plants, and serve to proofread the nascent product on the synthetic module [39]. They are specific for their corresponding ACP[40], [41]. Without a functioning TE2, erroneous acyl chains on a Pks module clog the machinery, decreasing product production by up to 90% [38]. If the Pks product is a secondary metabolite, this diminution is not crucial. However, in the case of mycolic acids, this diminution could render the bacterium non-viable.

**Figure 8:**
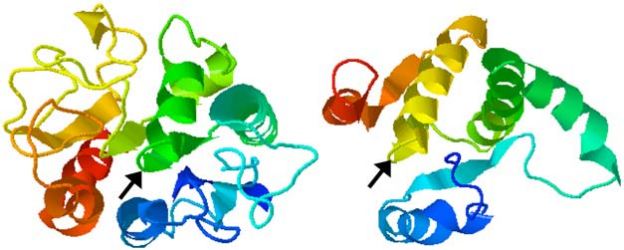
Structure prediction of Rv3802 using the Phyre server (http://www.sbg.bio.ic.ac.uk/~phyre/, Bennett-Lovsey et al, 2008). Model on left predicts a cutinase with 100% precision; modeling on right predicts a thioesterase with 70% precision. Black arrow represents location of catalytic serine 175.

A role as a TE1 or as a TE2 are both consistent with the putative essentiality of *Rv3802c* and the decrease in mycolic acids observed with THL. Significant amino acid similarity between TE1s or TE2s and cutinases is not found by search programs, yet the TE1s, TE2s and cutinases are all α/β-hydrolase fold enzymes with SDH motifs. In Rv3802, which otherwise shares little amino acid similarity to the cutinases (0–40%), the spacing of the SDH is more similar to thioesterases than to cutinases; structure prediction models find structural similarity of Rv3802 with known cutinases and thioesterases at 100% and 75% precision, respectively (Figure 8) [42].

Though there are multiple lines of evidence for involvement in mycolic acid synthesis, a drawback of our studies is that the data is indirect; further evaluation is underway, but due to complexity will take some time. The genetic location of *Rv3802c* and the thioesterase activity of its gene product are not likely to be coincidental, and inhibition by THL further supports this hypothesis. However, an alternative role interacting with host or mycobacterial lipids by Rv3802 or the other mycobacterial annotated cutinases is a possibility. These enzymes clearly possess PLA activity, and are able to hydrolyze the mycobacterial phospholipid PIM. If indeed there is a phospholipid carrier involved in mycolic acid synthesis, or if indeed host derived fatty acids can go directly into the FAS-II pathway, PLA activity may actually be linked to mycolic acid synthesis [7], [10].

### Conclusions

Our studies suggest *M. tuberculosis* Rv3802, previously annotated as a cutinase, is involved in mycolic acid synthesis. Activity as either a mycolyltransferase or a Pks associated thioesterase are logical roles for Rv3802, and are consistent with its genetic location, its discovered thioesterase activity, its inhibition by THL, and the previously published cell wall defects observed with use of THL as an antimycobacterial compound. However, this evidence is circumstantial and further direct evidence is needed to support or refute such a role for Rv3802.

## Materials and Methods

### Bacterial strains and culture conditions

*M. smegmatis* mc^2^ 155 (ATCC 700084) was purchased from ATCC. *M. smegmatis* was grown at 37°C in Luria-Bertani (LB) broth with 0.05% Tween80®. *Escherichia coli* strains DH5α and Rosetta 2 (DE3)pLysS (Merck) were grown at 37°C in LB broth. Antibiotics (Sigma) were added to media at the following concentrations: Ampicillan 100 µg/mL, Kanamycin 25–50 µg/mL, Chloramphenicol 34 µg/mL.

### Cloning Procedures

Standard PCR strategies with Taq DNA polymerase (Invitrogen) were used to amplify the *M. tuberculosis Rv3802c* and *M. smegmatis M_SMEG 1403* genes. PCR amplifications consisted of one cycle of denaturation (95°C, 10 min), followed by 30 cycles of amplification that included denaturation (95°C, 1 min), annealing (65°C, 1 min), and primer extension (72°C, 1 min). The primers used were as follows: 5′-catatgggatgccctgacctgcactggatcggc-3′ and 5′-gagctc **tcatttttcgaactgcgggtggctccaagcgct**gcctaacgtcggagcgggctc-3′ for *M. smegmatis MSMEG_1403* gene, and 5′-catatgggcgaatcgccgcccagcgcgg-3′ and 5′-gagctc **tcatttttcgaactgcgggtggctccaagcgctcct**atgtttggggtggggcgc-3′ for *M. tuberculosis Rv3802c* gene. Primers were designed to provide PCR amplified fragments containing a C-terminal streptavidin tag (bold) and flanking *NdeI* and *SacI* restriction sites (underline). Fragments were cloned into PCR2.1 (Invitrogen) and colonies for subsequent cloning were confirmed with sequencing. Correct clones were sub cloned into pET23a (Novagen). This vector was used as a template to make three mutants in *M. tuberculosis Rv3802c* via the QuickChange® II method (Stratagene, 2005245, per manufacturer protocol). Primers used were 5′-ctgatcgggtttgagcagggcgcggtg-3′ and 5′-caccgcgccctgctcaaacccgatcag-3′ for Rv3802 ΔS175E, 5′-ggaacctgggaggaatcgccgcagcag-3′ and 5′-ctgctgcggcgattcctcccaggttcc-3′ for Rv3802 ΔS86E, and 5′-cctgggagtcagagccgcagcagaaccc-3′ and 5′-gggttctcgtgcggctctgactcccagg-3′ for Rv3802 ΔS87E.

### Expression and purification of recombinant proteins *M. tuberculosis* Rv3802, its mutants, and *M. smegmatis* mc^2^155 MSMEG_1403 in *E. coli*

The constructs in pET23a described above were transformed into electrocompetent Rosetta 2 (DE3)pLysS for expression using the T7 promoter. Cultures were induced with 1 mM isopropyl-B-D-thiogalactopyranoside (IPTG) for 3 H at 37°C. Following induction, cell pellets were collected and re-suspended in 5 mL Strep-tactin lysis buffer (50 mM NaH_2_PO_4_ and 300 mM NaCl, pH 8.0 with 1 mg/mL lysozyme and 5 µg/mL DNase I) per 200 mL original induction culture, and stored at −20°C overnight. Thawed resuspensions with 2% (w/v) n-lauryl sarcosine was mixed for ≥60 min at room temperature until lysate was clear of large clumps. After centrifugation, target protein was purified from lysate using strep-tactin Superflow resin (IBA, Germany) per company protocol. Protein concentration and absorbance spectra of these aliquots were evaluated at 280 nm on a Nanodrop™ system. All eluted protein samples, if not used immediately, were stored at −70°C. Selected fractions were also analyzed with sodium dodecyl sulfate-polyacrylamide gel electrophoresis and either stained or immunoblotted using an anti-streptavidin tag antibody (IBA) at 1∶1500 and secondary horseradish peroxidase goat anti-mouse (1∶20 K), and visualized with chemiluminescence. Fractions were estimated to be at least 95% pure using SYPRO orange staining (molecular probes S5692, per protocol, sensitive to 5 ng of protein) with phosphoimager detection.

### Whole Cell Radiolabeling Experiments

For radiolabeling of whole *M. smegmatis* mc^2^155 cells with ^14^C, *M. smegmatis* was grown in LB medium supplemented with 0.05% Tween® 80 and, when in log phase, ^14^C-acetate (ARC 0173B) was added at 1 µCi/mL and cells were grown for four additional H. Cells were then pelleted, and extracted with 30 mL CHCl_3_∶CH_3_OH (1∶2, v/v) per gram of wet cells. Debris was again pelleted, extract removed, and re-extracted with the same volume of CHCl_3_∶CH_3_OH (2∶1, v/v). Extracts were combined and Folch washed. Organic layer was aliquoted and dried down in pre-weighed vials. For mass spectrometry, a parallel experiment was done without ^14^C-acetate. To isolate PIM species, 400 µg was resuspended in CHCl_3_ and applied to a silica column (Waters, WAT023537) and eluted with CHCl_3_∶CH_3_OH, in percentages (v/v) of 90∶10, 80∶20, 70∶30, 60∶40 and 50∶50. PIMs were collected from the 60∶40 and 50∶50 fractions.

### Mass spectrometry of extracted cell wall lipids

Cell wall lipids were isolated as above. Lipids were applied to Silica gel 60Å plates (Whatman, 4807-425), and developed in CHCl_3_∶CH_3_OH∶NH_4_OH (80∶20∶2, v/v/v). Individual bands of lipid were scraped from the plate into columns made from Pasteur pipettes that were fitted with 0.2 µm, Type FG Fluoropore membrane filters (Millipore). The silica gel was rinsed with 1 mL of 2∶1 *i*-C_3_H_7_OH/H_2_O and lipid species were eluted with 1 mL of 2∶1 (v/v) CHCl_3_/CH_3_OH. Samples were evaporated to dryness, re-dissolved in 2 µL of the same solvent and 0.5 µL was deposited onto a sample stage that had been pre-spotted with 2,5-dihydroxybenzoic acid (DHB) for analysis by matrix assisted laser desorption ionization (MALDI) mass spectrometry. Mass spectra using a laser wavelength of 337 nm were obtained from *m/z* 100 to 2400 using a QSTAR XL tandem mass spectrometer (Applied Biosystems/MDS Sciex, Thornhill, Ontario, Canada). The mass analyzer configuration of this instrument, mass-resolving quadrupole – collision quadrupole – orthogonal time-of-flight (Q-q-oTOF), allowed both full and collision induced dissociation (CID) spectra to be obtained. PIM species were identified based on molecular weight, interpretation of CID spectra, and comparison of spectra with data found in the literature [43], [44].

### Cell-free Assay on *M. smegmatis* crude cell wall and purified PIM components

Aliquots of ^14^C-radiolabeled cell wall or purified PIM were resuspended at 2 mg/mL in 50 mM Bis-tris, pH 7, with 0.25% Triton®-X 100. 50 µL of this was added to 10–50 µg of enzyme in 50 µL, for a total reaction volume of 100 µL. After 2–5 H, the reaction was quenched with 200 µL of CHCl_3_∶CH_3_OH∶HCl (50∶50∶0.3, v/v/v), and the organic phase removed and plated under a nitrogen stream on Silica gel 60Å plates (Whatman, 4807-425). Plates were developed in CHCl_3_∶CH_3_OH∶NH_4_OH (80∶20∶2, v/v/v) and visualized with a phosphoimager (radiolabel) and/or cupric sulfate or Dittmer-Lester stains (radiolabeled and unlabeled). For mass spectrometric analysis, a parallel experiment was performed without radiolabel and lipid species were recovered as described above.

### Determination of activity of recombinant proteins *M. tuberculosis* Rv3802, its mutants, and *M. smegmatis* mc^2^155 MSMEG_1403 on other phospholipid substrates

#### Control enzymes

6 units of snake venom PLA_2_ (*Naja mossambica mossambica*, Sigma P-7778) were used per reaction for a PLA activity control. Lyophilized *F. solani* cutinase (kind gift from Unilever Research and Development, Vlaardingen, The Netherlands) was resuspended at 3 mg/mL in H_2_O and then further diluted depending on assay.

#### Phospholipid assays

Unlabeled dipalmitoyl phosphatidylcholine (DPPC) was resuspended at 23 mM in chloroform, and used as a micellar backbone in all reactions. Radiolabeled phospholipids were as follows: phosphatidylcholine L-a-dipalmitoyl [dipalmitoyl-1-^14^C] (NEC682, 110 mCi/mM), bovine sphingomyelin [choline-methyl-^14^C] (NEC663, 52 mCi/mM), and phosphatidylethanolamine, L-a-palmitoyl-2-arachidonyl [arachidonyl-1-^14^C] (NEC-783, 48 mCi/mM), all from NEN Life Science Products, and 3-phosphatidyl-[3-^14^C]serine 1,2-dioleoyl (CFA757, 54 mCi/mM) from Amersham Biosciences. Cold DPPC with 0.1 µL of ^14^C-radiolabeled phospholipid per reaction were dried down and then resuspended with 50 µL of 50 mM bis-tris pH 7.4 buffer with 0.25% Triton®-X 100 per reaction to make mixed micelles. This solution was sonicated 30 s×3, vortexing in between. 50 µL of this substrate was then added to 50 µL of enzyme solution (10–50 µg), and incubated at 37°C three H. The reaction was extracted with 100 µL of chloroform/methanol/HCl (50∶50∶0.3, v/v/v), vortexed and centrifuged at 13,000 rpms for two min. The organic phase was then plated on 60 Å silica plates (Whatman 4807-425) and then developed in CHCl_3_∶CH_3_OH∶H_2_O (v/v/v, 14∶6∶1) solvent. Lipids were visualized with a phosphoimager.

### Thioesterase, esterase, and transferase activity of recombinant of *M. tuberculosis* Rv3802, its mutants, and *M. smegmatis* mc^2^155 MSMEG_1403, with and without the inhibitor THL

Control enzymes included *F. solani* cutinase, the general esterase *Thermomyces lanuginosus* (Sigma L-0777) and *M. tuberculosis* Ag85C (Tuberculosis Research Materials and Vaccine Testing, Colorado State University).

#### Nitrophenyl ester assays

hydrolysis of NPB (Sigma, N9876) was used as a surrogate for cutinase activity. Though both *M. smegmatis* MSMEG_1403 and *M. tuberculosis* Rv3802 were active in this assay, kinetics was done with only Rv3802. For kinetic calculations, each 200 µL reaction contained 0.01 mg of enzyme (1.53 µM), the path length was 0.8 cm and the extinction coefficient was 15250 M^−1^ cm^−1^ [45]. The NPB 5.69 M stock was diluted in 50 mM bis-tris/5% glycerol (pH 7.0) for use in the assays. The final substrate concentration ranged from 1.56 mM–200 mM. Assays were done in 96 well plates, in triplicate, at 37°C for four H, with proper controls. There were three replicate experiments. Absorbance was read by a plate spectrophotometer at 415 nm at various time points. Prizm 5.0 software were used to determine K_m_ and V_max_ and these were used to determine the kcat and specificity constant for the esterase activity of Rv3802 and mutants. Up to 10 units of snake venom PLA was also tested and was inactive.

#### Thioesterase activity assays

hydrolysis of P-CoA (Avanti, 870716) and D-CoA, (Sigma, D5269) were used to measure thioesterase activity. When the thioester bond is hydrolyzed, the free sulfur on CoA is attacked by 5,5′-dithio-bis(2-nitrobenzoic acid) (DTNB), which releases a measurable nitrophenyl group, 5-thio-2-nitrobenzoate (TNB^2−^). For kinetic calculations, each 200 µL reaction contained 3 µg of enzyme (461 nm), the path length was 0.8 cm and the extinction coefficient was 14150 M^−1^ cm^−1^ for TNB^2−^ (Sigma). The P-CoA 20 mM stock was diluted in 50 mM bis-tris (pH 7.0) for use in the assays. The DTNB 40 mM stock was diluted in EtOH to give a molar equivalent amount of DTNB to acyl-CoA for the reaction (Increasing DTNB relative to P-CoA did not increase reaction rate, and DTNB did not react with enzyme alone). The final P-CoA concentration ranged from 4.875 µM – 2.5 mM, and the final D-CoA concentration ranged from 0.078 to 5.0 mM. Assays were done in 96 well plates, in triplicate, at 37°C for three H, with proper controls. There were four replicate experiments. Absorbance was read by a plate spectrophotometer at 415 nm at various time points. Prizm 5.0 software was used to determine K_m_ and V_max_ and these were used to determine the kcat and specificity constant for the thioesterase activity of Rv3802. For radiolabel ^14^C-P-CoA assays, protocol was followed as in ^14^C-phospholipids assays above.

#### THL inhibition assays

four concentrations of THL (Sigma, 04139) were selected in order to determine K_i_ and nature of inhibition of Rv3802, based on pilot experiments: 1.56 µM, 0.78 µM, 0.39 µM, and 0.195 µM. 100% inhibition of activity was seen at 3 µM final concentration. Inhibition assays were performed identically to the thioesterase assays without inhibitor described previously, except that after the addition of THL and before the addition of P-CoA, the assay was incubated at 37°C for 30 min [39]. The Reaction was run for 1–2 H, in duplicate, and four replicates were performed. Changes in K_m_ and V_max_ (compared to non-inhibited Rv3802) were determined using Prizm 5.0 software, as were apparent K_i_ and IC50 values. For inhibition of *M. smegmatis* MSMEG_1403, each 200 µL reaction contained 0.3 µg (0.495 µM). THL concentration was increased to a maximum of 25 µM (a molar excess compared to enzyme of 505) in an attempt to detect inhibition.

#### Transferase assays

donors and recipients were paired for various transferase assays to assess whether the purified enzymes could hydrolyze a known substrate and transfer it to an acceptor. Assays included 10–50 µg of either Rv3802 or MSMEG_1403 protein, and were incubated for 1–72 H. Pairings included: 1) ^14^C-trehalose (0.25 µCi) with crude cell wall lipids (50 µg) from *M. tuberculosis*; 2) ^14^C-P-CoA (0.25 µCi) as donor with mannose-6-phosphate (1–10 mM) or glycerol-6-phosphate (1 mM) as acceptor with crude cell wall extract (proteins and lipids for unknown cofactors or other acceptors, total 0.1 to 1 mg, made per previously described protocol[27]; 3) same as two with addition of crude cytosolic extract, 4) same as in two and three with addition of ^14^C-trehalose (0.25 µCi); 5) same as two and three with ^14^C-DPPC instead of ^14^C-P-CoA. As a positive control for the mycolyltranferase assays using ^14^C-trehalose as an acceptor, Ag85C was used at 10–50 µg per assay.
